# The Erect Posture for Gynecological Examination

**Published:** 1894-07

**Authors:** 


THE ERECT POSTURE FOR GYNECOLOGICAL EXAMINATION.—Wm. B. Dewees (*Medical and Surgical Reporter*, December 2, 1893,) urges the importance of examining women in the erect posture, particularly when it is desired to accurately determine the nature and extent of displacements of the uterus or its appendages, as these conditions are apt to be more troublesome to the woman while on her feet, and are not so marked when she assumes the recumbent posture; and, therefore, if the examination is made with the patient in any other than the erect position, a true estimate of the defect cannot be obtained. Also, a pessary which may appear to support the parti-wall while the patient is in the recumbent posture, may be found to be of no value when the erect posture is assumed. Other conditions for which this method of examination is advised are: Vesical and rectal disorders, lack of perinea and vaginal support, ovarian and tubal diseases, abdominal and pelvic tumors, differentiation between abdominal tumors and pregnancy. —(*Ex.*)

